# Supplementary material for: Identification of CAMTA Gene Family in Heimia myrtifolia and Expression Analysis under Drought Stress
Source: Plants (Basel). 2022 Nov 9;11(22):3031. doi: 10.3390/plants11223031 (PMC9698416; doi:10.3390/plants11223031)
Supplement: Supplementary file 1 [file plants-11-03031-s001.zip › plants-1957460-supplementary.pdf]

Table S1. Basic information on the multi-species CAMTA family

Category	Species	Protein name	Accession numbers	Lengths of the proteins / aa
Eudicots	*Arabidopsis thaliana*	AtCAMTA1	Q9FY74	1007
	A. thaliana	AtCAMTA2	Q6NPP4	1050
	A. thaliana	AtCAMTA3	Q8GSA7	1032
	A. thaliana	AtCAMTA4	Q9FYG2	1016
	A. thaliana	AtCAMTA5	O23463	923
	A. thaliana	AtCAMTA6	Q9LSP8	838
	Nicotiana tabacum	NtCAMTA1	XM_016579639.1	968
	N. tabacum	NtCAMTA2	XM_016597284.1	1107
	N. tabacum	NtCAMTA3	XM_016597288.1	1106
	N. tabacum	NtCAMTA4	XM_016597294.1	1086
	N. tabacum	NtCAMTA5	XM_016608516.1	1049
	N. tabacum	NtCAMTA6	XM_016616857.1	791
	N. tabacum	NtCAMTA7	XM_016622590.1	1103
	N. tabacum	NtCAMTA8	XM_016622591.1	1102
	N. tabacum	NtCAMTA9	XM_016630378.1	1056
	N. tabacum	NtCAMTA10	XM_016630379.1	1055
	N. tabacum	NtCAMTA11	XM_016633652.1	875
	N. tabacum	NtCAMTA12	XM_016635831.1	901
	N. tabacum	NtCAMTA13	XM_016636014.1	926
	N. tabacum	NtCAMTA14	XM_016636015.1	826
	N. tabacum	NtCAMTA15	XM_016644851.1	926
	N. tabacum	NtCAMTA16	XM_016651473.1	969
	N. tabacum	NtCAMTA17	XM_016652305.1	965
	N. tabacum	NtCAMTA18	XM_016652306.1	962
	N. tabacum	NtCAMTA19	XM_016652307.1	913
	Punica granatum	PgCAMTA1	MTKT01005898.1	1016
	P. granatum	PgCAMTA2	MTKT01005898.1	1294
	P. granatum	PgCAMTA3	MTKT01005569.1	1078
	P. granatum	PgCAMTA4	MTKT01004486.1	922
	P. granatum	PgCAMTA5	MTKT01003224.1	934
	P. granatum	PgCAMTA6	MTKT01000799.1	892
	Solanum lycopersicum	SlCAMTA1	Solyc01g057270.2.1	1037
	S. lycopersicum	SlCAMTA2	Solyc01g060170.2.1	1049
	S. lycopersicum	SlCAMTA3	Solyc01g105230.2.1	920
	S. lycopersicum	SlCAMTA4	Solyc04g056270.2.1	939
	S. lycopersicum	SlCAMTA5	Solyc05g015650.2.1	910
	S. lycopersicum	SlCAMTA6	Solyc12g035520.1.1	1097
	S. lycopersicum	SlCAMTA7	Solyc12g099340.1.1	916
	Populus trichocarpa	PtCAMTA1	Potri.001G057800.1	998
	P. trichocarpa	PtCAMTA2	Potri.001G057800.2	967
	P. trichocarpa	PtCAMTA3	Potri.003G170600.1	981

	P. trichocarpa	PtCAMTA4	Potri.003G170600.2	916
	P. trichocarpa	PtCAMTA5	Potri.005G075100.1	1117
	P. trichocarpa	PtCAMTA6	Potri.005G075100.2	1105
	P. trichocarpa	PtCAMTA7	Potri.005G075100.3	992
	P. trichocarpa	PtCAMTA8	Potri.007G093400.1	1092
	P. trichocarpa	PtCAMTA9	Potri.007G093400.2	1091
	P. trichocarpa	PtCAMTA10	Potri.007G093400.3	1021
	P. trichocarpa	PtCAMTA11	Potri.008G107900.1	908
	P. trichocarpa	PtCAMTA12	Potri.008G107900.2	846
	P. trichocarpa	PtCAMTA13	Potri.010G141700.1	916
	P. trichocarpa	PtCAMTA14	Potri.010G153100.1	1000
	P. trichocarpa	PtCAMTA15	Potri.010G153100.2	965
	P. trichocarpa	PtCAMTA16	Potri.010G153100.3	1000
	P. trichocarpa	PtCAMTA17	Potri.010G153100.4	992
	P. trichocarpa	PtCAMTA18	Potri.010G153100.5	990
	Zea mays	ZmCAMTA1	ZEAMMB73_Zm00001d028007	1026
	Z. mays	ZmCAMTA2	ZEAMMB73_Zm00001d030518	1019
	Z. mays	ZmCAMTA3	ZEAMMB73_Zm00001d003958	980
	Z. mays	ZmCAMTA4	ZEAMMB73_Zm00001d014286	1021
	Z. mays	ZmCAMTA5	ZEAMMB73_Zm00001d021516	1013
	Z. mays	ZmCAMTA6	ZEAMMB73_Zm00001d047209	1034
Monocots	*Z. mays*	ZmCAMTA7	ZEAMMB73_Zm00001d025235	977
	Oryza sativa	OsCAMTA1	CM000126.1	878
	O. sativa	OsCAMTA2	CM000128.1	989
	O. sativa	OsCAMTA3	CM000128.1	1031
	O. sativa	OsCAMTA4	CM000129.1	915
	O. sativa	OsCAMTA5	CM000132.1	829
	O. sativa	OsCAMTA6	CM000132.1	985
	O. sativa	OsCAMTA7	CM000135.2	995
Bryophyte	*Physcomitrella patens*	PpCAMTA1	PNR58426	1155
	P. patens	PpCAMTA2	PNR54912	1186
	Selaginella moellendorffii	SmCAMTA1	EFJ26780	982
	S. moellendorffii	SmCAMTA2	EFJ26472	982
	S. moellendorffii	SmCAMTA3	EFJ09518	517
Fern	*S. moellendorffii*	SmCAMTA4	EFJ36306	543
	S. moellendorffii	SmCAMTA5	EFJ06785	625
	S. moellendorffii	SmCAMTA6	EFJ21743	625
	S. moellendorffii	SmCAMTA7	EFJ28494	932
	S. moellendorffii	SmCAMTA8	EFJ30381	917
	Amborella trichopoda	AmtCAMTA2	ERN14613	1136
Basal angiosperm	*A. trichopoda*	AmtCAMTA3	ERN01955	1091
	A. trichopoda	AmtCAMTA4	ERN09861	970
	A. trichopoda	AmtCAMTA5	ERN16758	909
Algae	*Micromonas commoda*	McCAMTA1	ACO64707	1564
